# Wetland characteristics linked to broad-scale patterns in *Culiseta melanura* abundance and eastern equine encephalitis virus infection

**DOI:** 10.1186/s13071-017-2482-0

**Published:** 2017-10-18

**Authors:** Nicholas K. Skaff, Philip M. Armstrong, Theodore G. Andreadis, Kendra S. Cheruvelil

**Affiliations:** 1 Department of Fisheries and Wildlife, Michigan State University, East Lansing, MI USA; 2 Ecology, Evolutionary Biology & Behavior Program, Michigan State University, East Lansing, MI USA; 3 Department of Environmental Sciences, The Connecticut Agricultural Experiment Station, New Haven, CT USA; 4 Lyman Briggs College, Michigan State University, East Lansing, MI USA

**Keywords:** Eastern equine encephalitis virus, Wetlands, *Culiseta melanura*, Vegetation, Connectivity, Hydrology, Drought

## Abstract

**Background:**

Eastern equine encephalitis virus (EEEV) is an expanding mosquito-borne threat to humans and domestic animal populations in the northeastern United States. Outbreaks of EEEV are challenging to predict due to spatial and temporal uncertainty in the abundance and viral infection of *Cs. melanura*, the principal enzootic vector. EEEV activity may be closely linked to wetlands because they provide essential habitat for mosquito vectors and avian reservoir hosts. However, wetlands are not homogeneous and can vary by vegetation, connectivity, size, and inundation patterns. Wetlands may also have different effects on EEEV transmission depending on the assessed spatial scale. We investigated associations between wetland characteristics and *Cs. melanura* abundance and infection with EEEV at multiple spatial scales in Connecticut, USA.

**Results:**

Our findings indicate that wetland vegetative characteristics have strong associations with *Cs. melanura* abundance. Deciduous and evergreen forested wetlands were associated with higher *Cs. melanura* abundance, likely because these wetlands provide suitable subterranean habitat for *Cs. melanura* development. In contrast, *Cs. melanura* abundance was negatively associated with emergent and scrub/shrub wetlands, and wetland connectivity to streams. These relationships were generally strongest at broad spatial scales. Additionally, the relationships between wetland characteristics and EEEV infection in *Cs. melanura* were generally weak. However, *Cs. melanura* abundance was strongly associated with EEEV infection, suggesting that wetland-associated changes in abundance may be indirectly linked to EEEV infection in *Cs. melanura*. Finally, we found that wet hydrological conditions during the transmission season and during the fall/winter preceding the transmission season were associated with higher *Cs. melanura* abundance and EEEV infection, indicating that wet conditions are favorable for EEEV transmission.

**Conclusions:**

These results expand the broad-scale understanding of the effects of wetlands on EEEV transmission and help to reduce the spatial and temporal uncertainty associated with EEEV outbreaks.

**Electronic supplementary material:**

The online version of this article (10.1186/s13071-017-2482-0) contains supplementary material, which is available to authorized users.

## Background

Eastern equine encephalitis virus (EEEV; *Togaviridae*, *Alphavirus*) is a highly pathogenic mosquito-borne zoonosis that is responsible for severe disease in humans and equines, resulting in high mortality and severe neurological impairment in most survivors [1–4]. Outbreaks of EEEV in the northeastern United States occur intermittently [2], but appear to be resurging and expanding throughout the region [3, 4]. The frequency and intensity of these events are difficult to predict due to spatial and temporal uncertainty in the population abundance and viral infection rate of the primary vector species, *Culiseta melanura* [5–7]. Abundance and EEEV infection of *Cs. melanura* may be closely linked to local land cover characteristics, particularly wetland cover that provides important habitat for vectors and susceptible hosts [6, 8–10]. Therefore, increasing our understanding of wetland effects on *Cs. melanura* abundance and viral infection can help to identify key elements that enhance potential outbreaks.

The influence of wetlands on *Cs. melanura* and EEEV infection may depend on the spatial scales and temporal periods over which relationships are evaluated. The effects of wetlands may change in magnitude or direction depending on the spatial scales of analysis, and temporally dynamic drivers may have lagged effects because precursors to shifts in mosquito infection and abundance can arise months in advance [11, 12]. The scales and lags most predictive of *Cs. melanura* abundance and EEEV infection may correspond with the distance and timing of vector and host movements [13]. However, uncertainty surrounding current estimates and the relative importance of vector and hosts movement necessitates evaluating the effects of wetland cover and temporally-varying wetland conditions at a range of potential spatial scales and temporal lags [12, 14].

To date, knowledge of the potential impacts of wetland characteristics on EEEV transmission in the northeastern US is derived from studies using two methodologies. There are small-scale studies that focus on *Cs. melanura* habitat utilization in individual wetlands, but do not estimate *Cs. melanura* habitat utilization at broad epidemiologically relevant scales [6, 8, 15]. There are also studies evaluating *Cs. melanura* abundance at the broad spatial scales most relevant to pathogen spread and management. However, these studies typically aggregate wetlands of various vegetation, structural, and inundation types into just one or a few categories. This fails to account for the important differences in wetland habitats that have been identified in small-scale studies [9, 16–18]. Based on the results of these analyses, there is evidence that the specific vegetative, structural, and inundation characteristics of wetlands may have important effects on both *Cs. melanura* population abundance and EEEV infection at broad scales.

Wetland characteristics can influence either *Cs. melanura* abundance or infection with EEEV. For our analyses of *Cs. melanura* abundance, we focus on three wetland characteristics that may have important effects - wetland vegetation, wetland connectivity, and wetland inundation classification:

(i) Wetland vegetation: Vegetation imposes important constraints on the suitability of wetland habitats to *Cs. melanura* larval development. Studies have found that larval *Cs. melanura* proliferate in freshwater forested swamps, both deciduous and evergreen, usually in recesses beneath tree-roots and other dark, thermally stable microhabitats [8, 15, 19]. These precipitation- and groundwater-fed wetlands are dominated by red maple (*Acer rubrum*), Atlantic white cedar (*Thuja occidentalis*), yellow birch (*Betula alleghaniensis*), and eastern hemlock (*Tsuga canadensis*) [2, 10, 20]. Wetlands dominated by different vegetation types, like emergent and scrub-shrub vegetation, appear to be of minor importance as *Cs. melanura* larval habitats [9, 20].
(ii) Wetland connectivity: Connectivity of wetlands to other aquatic habitats may influence mosquito abundance via changes in mosquito predator dispersal. Mosquito predators typically include water-dependent taxa like amphibians, small fishes, and larval macroinvertebrate insects [20, 21]. Studies of several mosquito species, although not *Cs. melanura*, have found that the density and proximity of wetland habitats and the number of stream connections between aquatic environments have a positive influence on mosquito predator movement. These forms of connectivity help to stabilize predator metacommunities leading to larger predator populations and potential reductions in mosquito abundance [22–24].
(iii) Wetland inundation classification: A wetland’s inundation classification (a way of categorizing wetlands based on the duration and timing of surface inundation) in conjunction with changes in hydrological wetness (water table depth) may directly or indirectly influence *Cs. melanura* abundance. Drying and subsequent re-inundation of semi-permanently inundated wetlands have been indirectly linked to increases in larval mosquito biomass (although not tested in *Cs. melanura*) via decreases in mosquito predator biomass [21]. In contrast, hydrological changes in wetlands with other inundation classifications (i.e. permanent and temporary wetlands) have limited effects on mosquito biomass [21]. Finally, increases in groundwater levels, leading to sustained inundation of larval habitats during the winter and transmission season, can stabilize larval habitat and directly promote larger populations of *Cs. melanura* [2, 6].

The above relationships are primarily derived from local-scale studies linking wetland characteristics and larval abundance. It is unclear whether these results apply to broad-scale patterns in adult *Cs. melanura* abundance or whether findings derived from other mosquito species apply to *Cs. melanura*.

Wetlands may also influence EEEV infection in *Cs. melanura* via three wetland characteristics: wetland vegetation, wetland size, and wetland inundation classification:

(i) Wetland vegetation: Wetland vegetation imposes constraints on the locations where contact between vectors and susceptible avian hosts occurs. Forest wetlands are key sources of food, water, and nesting habitat for a variety of passerine species (primary avian EEEV hosts) and support double the bird density of nearby upland sites [10]. Although recently-emerged *Cs. melanura* adults disperse from wetland habitats [6, 19], vectors will opportunistically feed on nearby hosts if they are in high densities [25, 26]. Therefore, forested wetlands and their surroundings may be focal points for contact between susceptible passerines and *Cs. melanura*, leading to an increase in vector EEEV infection.
(ii) Wetland size: Wetland size may affect mosquito infection due to the positive relationship between wetland size and avian species richness ([27, 28] reviewed in [29]). Previous studies have shown that small wetlands tend to have low avian species richness and to be dominated by highly susceptible passerines, whereas large wetlands have a diverse mix of susceptible and non-susceptible birds that reduces viral amplification (though not tested in EEEV system) [27, 28].
(iii) Wetland inundation classification: A wetland’s inundation classification combined with changes in hydrological wetness (water table depth) may alter host density and mosquito infection. Research on West Nile virus (WNV) and St. Louis encephalitis virus (SLEV) indicates that drought-driven reductions in water table depth reduces the availability of aquatic habitats forcing vectors and avian hosts onto a few remaining aquatic refuges [30–34]. This may result in increased contact between important vectors and hosts and subsequent increases in vector infection.

To assess the influence of broad-scale wetland characteristics on EEEV transmission, we investigated links between wetland land cover characteristics and *Cs. melanura* abundance and EEEV infection in Connecticut. We focus our analyses on wetlands with four vegetation types: (i) evergreen forest; (ii) deciduous forest; (iii) emergent vegetation; and (iv) scrub-shrub vegetation. We hypothesize that forested wetland habitats will be positively associated with *Cs. melanura* abundance and EEEV infection, and that there will be no strong relationships between emergent and scrub-shrub wetlands and both response variables.

Further, we examine several other wetland characteristics with potential influences on EEEV transmission: (i) hydrological connectivity to streams; (ii) wetland patch size; and (iii) inundation classification and hydrological wetness. We hypothesize that connectivity will have a negative relationship with abundance, and that wetland size will have a positive relationship with EEEV infection. We also expect that drought in forested semi-permanent wetlands during the previous transmission season, and inundation during the winter and during an ongoing transmission season will be positively associated with *Cs. melanura* abundance. Further, we expect that drought during the transmission season be positively associated with EEEV infection. Finally, we examine the spatial scales over which these wetland characteristics are most important and both the spatial scales and monthly temporal lags associated with the strongest effects of wetland inundation and hydrological wetness conditions. Overall, we aim to identify landscape-level wetland effects on *Cs. melanura* abundance and EEEV infection, the spatial scales and temporal lags over which these effects are most important, and to ultimately help foster a better understanding of broad-scale patterns of EEEV transmission.

## Methods

### Mosquito trapping, identification, and viral isolation

**Fig. 1:**
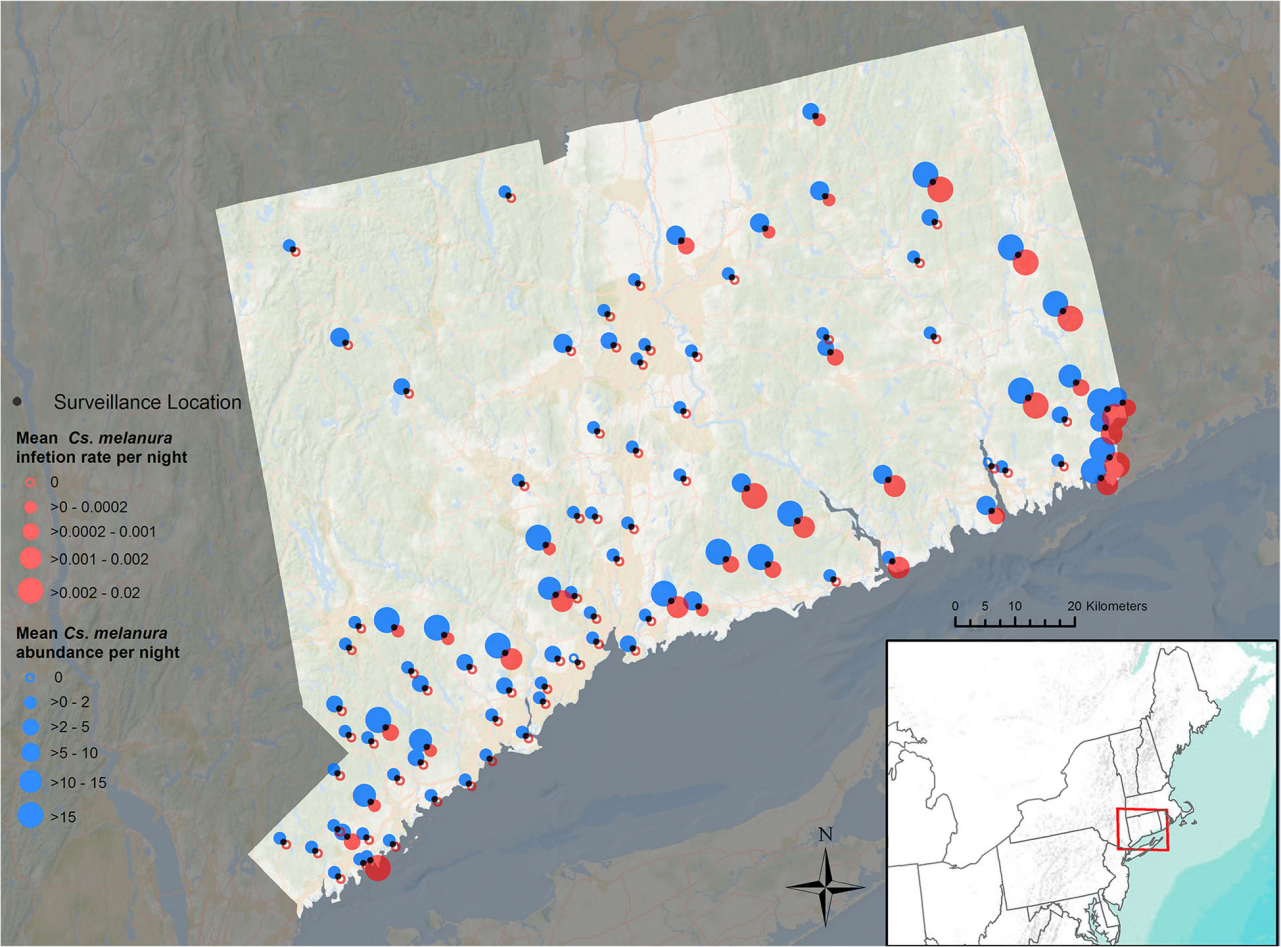
Study extent, sample locations, vector abundance and vector EEEV infection rate for all sampling years (2001–2014). Black points mark each of the mosquito trapping locations. The size of the blue circle above the black point represents mean *Cs. melanura* abundance per sampling night at the sampling location and the size of the red circle below the black point reflects the mean EEEV infection rate of *Cs. melanura* at the sampling location

Data were acquired across the state of Connecticut, in the northeastern United States (Fig. 1), which experienced EEEV infections in *Cs. melanura* in most years during the study period, 2001–2014 [35]. Mosquito trap data were collected as part of the State of Connecticut Mosquito Trapping and Arbovirus Testing Program [36, 37]. There were 91 trapping sites each year, but 97 total sites were used over the course of the entire study period because the locations of 6 traps changed over time. Traps were placed in a variety of habitat types ranging from urban to rural, and from upland to wetland habitats. They were deployed in the late morning or early afternoon and retrieved the following morning at least once every ten days. In most cases, two types of traps were used at each site, a CO_2_ (dry ice) - baited CDC miniature light trap with an aluminum dome, and a CDC gravid mosquito trap [38] baited with a rabbit chow infusion (Purina Mills LLC, St. Louis, MO, USA) [36].

Mosquitoes were identified to species and placed in pools ranging from 1 to 50 individuals. We included only *Cs. melanura* in our analyses, because it is the primary contributor to EEEV transmission in CT [5]. EEEV was identified from Vero cell positive cultures using either reverse transcription-polymerase chain reaction (RT-PCR) (2001) or TaqMan RT-PCR (2002–2014). Further details on sample site locations, trapping protocol, and viral isolation were previously described [36].

### Wetland cover data collection and processing

Circular buffers with radii of 50 m, 100 m, 200 m, 500 m, 750 m, 1000 m, 1500 m, 2000 m, 3000 m, 4000 m, and 5000 m were generated around each sampling site using ArcGIS 10.3.1. Using the same software, we calculated the proportion of the total buffer area covered by wetlands with the following characteristics: deciduous forest vegetation, evergreen forest vegetation, emergent vegetation, scrub/shrub vegetation, and forested wetlands with a semi-permanent inundation classification. We also calculated the average size of forested wetlands within each buffer, the average number of stream connections in forested wetlands within each buffer, and the area of forested semi-permanent wetlands relative to all other wetland types. This last variable was included in addition to the variable representing the proportional area of forested semi-permanent wetland within each buffer because it captures the effects of inundation-driven vector and host movement between wetland types.

Wetland data were acquired from geodatabases associated with LAGOS-NE [39, 40]. In particular, these geodatabases contained wetland location, size, vegetative cover, and inundation classification data originating from the National Wetlands Inventory (NWI) [41], which holds information on all aerially visible wetlands in the US (38,404 wetlands in CT). Wetland hydrological connectivity to streams was not included in the NWI, but was calculated and made available within LAGOS-NE using ArcGIS 10.1 and the LAGOS-GIS Toolbox [39, 42]. Stream locational data were acquired from 1:24,000 scale National Hydrography Dataset (NHD) [43]. Stream connections included NHD features defined as “Stream/River”, “Canal/Ditch”, “Pipeline”, or “Underground Conduit” [39, 43].

The mean quantity of impervious surface land cover was also calculated within each buffer for inclusion in models to control for the effects of human development on mosquito abundance and viral infection. Impervious surface data were acquired from the 2006 version of the National Land Cover Database (NLCD) percent developed imperviousness layer [44]. We used the 2006 version because it is closest to the midpoint of our study period and is likely the closest representation of the status of impervious surfaces over the entire time-period. The percent coverage of impervious surfaces was calculated by averaging the impervious surface percent values of all the raster pixels within each buffer.

### Hydrological wetness conditions data collection and processing

We quantified the monthly Palmer Hydrological Drought Index (PHDI) from 2000 to 2014 at each trapping location [45]. Because PHDI is a hydrological, rather than a meteorological measure of drought and wetness, it generally lags behind measures of precipitation and temperature and reflects groundwater levels. Therefore, it is likely a good estimate of the inundation status of the forested wetlands preferred by *Cs. melanura* [2]. PHDI data are available at the climate division scale, of which there are three in CT. We identified the climate division that contains each trapping site and attributed the PHDI data from that climate division to the trapping site. We then generated monthly PHDI lags by extracting PHDI data 0 to 12 months prior to the sampling month in 1-month increments (R 3.2.3).

### Spatial scale and monthly lag selection

Two response variables were included in our analyses: (i) monthly mean *Cs. melanura* abundance per night and (ii) monthly EEEV presence/absence in *Cs. melanura*. Response variables were aggregated by month because individual trapping days often yielded no virus isolations and the time between individual trapping days was not always constant. We determined monthly mean *Cs. melanura* abundance per night by calculating the mean number of mosquitoes collected per night at each sampling site every month. This metric combines the total capture of all traps, usually a gravid trap and a light trap, operated at each site. We determined the monthly presence/absence of EEEV by evaluating whether any pools had tested positive for EEEV over the course of a month at each sampling site. We identified the monthly lags and spatial scales for each covariate that best explain variation in these response variables by adapting a previously used approach [46], which we have fully described in Additional file 1. This method allowed us to avoid: (i) making the assumption that a variable’s best fitting scale or lag in a univariate model is the best scale/lag in the context of the full model and (ii) evaluating all possible combinations of scales and lags in the context of the full model, which is computationally impractical and prone to statistical error [46, 47].

### Analysis of wetland effects on *Cs. melanura* infection and abundance

Generalized Additive Models (GAMs) were used to evaluate and visualize the relationships between the response and explanatory variables (at the best performing scales/lags) using the *mgcv* package in R 3.2.3 [48]. GAMs are non-parametric models that build on Generalized Linear Models (GLMs) by loosening assumptions about the relationship between predictor and response variables. Whereas this relationship must be linear in GLMs, the only constraint in GAMs is that the relationship be smooth. GAMs include GLMs when the smoothing parameters are linear, but they also allow for non-linear relationships between predictors and response [49, 50]. We used these models to avoid the need to pre-specify the relationship between the predictors and each response (e.g. linear, quadratic, cubic etc.), because there is little preexisting information indicating the shape of these relationships [12, 51]. The *mgcv* package also includes a feature that automatically penalizes the smoothing parameters associated with each predictor variable so that predictors are removed from the model if they do not improve model fit and have smoothing parameters that approach infinity [52]. This selection tool indicated that our full models were also our most parsimonious, and this was further substantiated by assessments of AIC scores during the scale/lag selection process (Additional file 1), which indicated that all covariates measured at the selected scales/lags improved AIC scores by at least 2 units. A basis dimension of 3 or 4, corresponding to 2 or 3 degrees of freedom respectively, was used for all penalized regression smoothers to limit model over-fitting and yet accommodate parabolic and threshold relationships. The deviance explained by individual covariates was calculated using the *dev.expl.mgcv* package in R 3.2.3 [53].

We included additional non-wetland variables in our models in order to control for their effects. We included the percent of impervious surface coverage (many potential effects on vector and host populations), the month when sampling occurred (to account for within-season temporal autocorrelation), and the number of gravid traps relative to the number of light traps (1 of each type was not always deployed each night). We also included the mean abundance of *Cs. melanura* captured per night in the EEEV infection models in order to control for the potential effect of the number of mosquitoes captured on the likelihood of EEEV detection and simultaneously to assess whether abundance influences viral amplification (although we are unable distinguish between these possibilities). Finally, we assessed several model assumptions including concurvity, the GAM equivalent of collinearity between covariates, and observed nothing problematic. We did not account for spatial autocorrelation in our models because Moran’s I analyses on model residuals indicated that spatial autocorrelation was not statistically significant in any of our best performing models.

## Results

### Mosquito collection and virus isolation

Mosquito collection data summarized across the 13-year study period and all sampling sites are presented in Table 1. A total of 125,955 individual *Cs. melanura* were collected. Abundance was highest June through August, tapered off slightly in September, and was very low by October. In contrast, the proportion of EEEV positive pools was high in September and October, slightly lower in August, and near zero in June and July (Table 1). The greatest abundances of *Cs. melanura* were detected at sampling sites in the southern and eastern parts of Connecticut; these areas also coincided with the highest mean EEEV infection rates, with some exceptions (Fig. 1).

**Table 1:** Summary of *Cs*. *melanura* abundance and EEEV infection data. Data are aggregated across all study years (2001–2014) and all 97 sampling sites

Month	Total captured	Mean captured/night ± SE	# Positive pools	% Pools positive
June	30,376	8.66 ± 0.38	0	–
July	30,467	8.45 ± 0.57	2	0.1
August	32,563	7.32 ± 0.47	49	1.9
September	27,947	6.00 ± 0.32	129	5.2
October	6006	1.56 ± 0.13	41	3.5

*Abbreviation*: *SE* standard error

### Optimum spatial scale and temporal lag of wetland and hydrological effects

We identified key monthly lags in hydrological wetness conditions (PHDI) and key spatial scales over which wetlands were most closely associated with *Cs. melanura* abundance and EEEV presence/absence. We present those results below for each response variable.

#### *Culiseta melanura* abundance

**Fig. 2:**
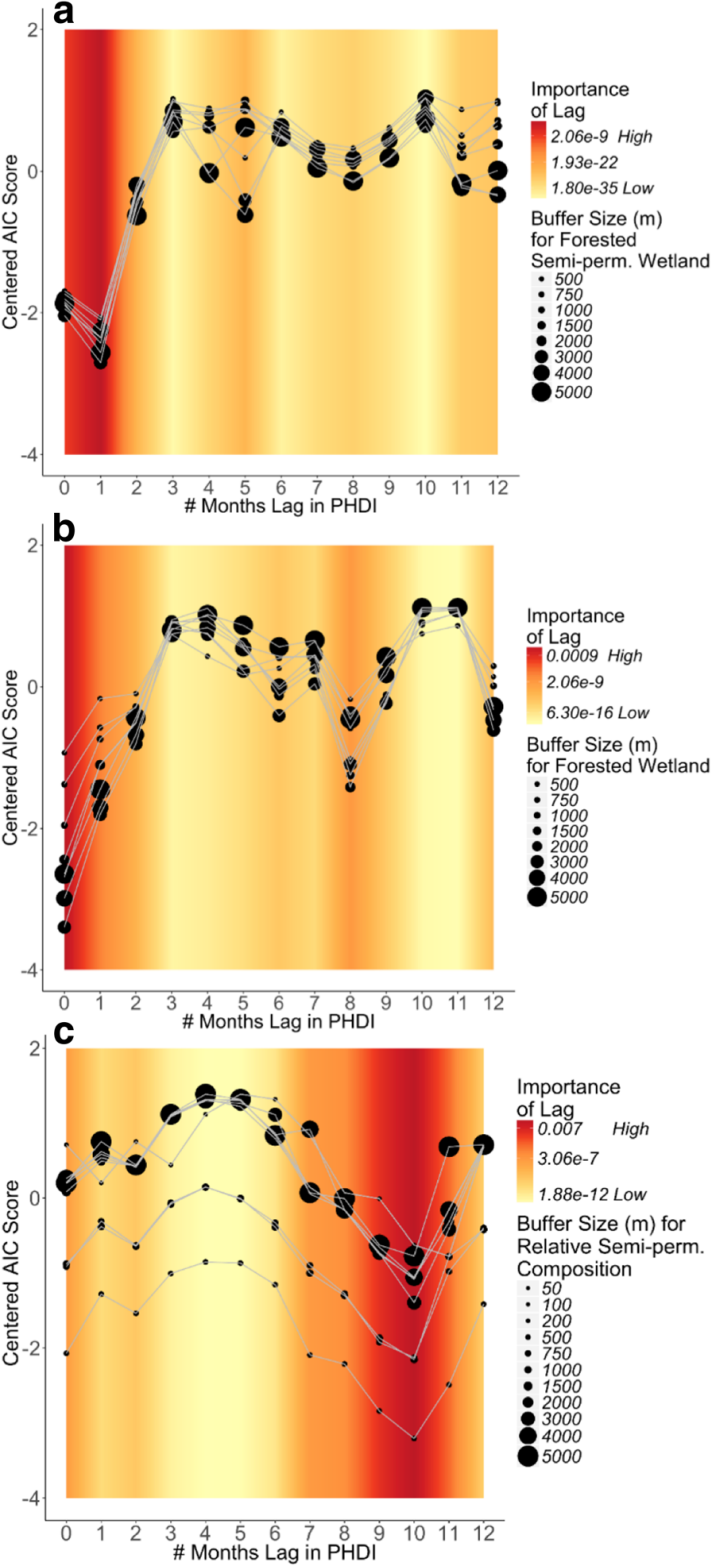
Relative importance of spatial scales and temporal lags. Each plot highlights the particular monthly lags in hydrological wetness conditions (PHDI) and the spatial scales (for wetland variables) that are included in the best performing models (determined by AIC). Each black point represents a different model explaining either *Cs. melanura* abundance (**a**, **b**) or EEEV presence/absence (**c**). Each model has a unique combination of spatial scale for the wetland variable (represented by point size) and monthly lag in hydrological conditions (PHDI; x-axis value). A different wetland variable is included in each plot: (**a**) forested semi-permanent wetlands; (**b**) forested wetlands; and (**c**) the abundance of forested semi-permanent wetland relative to other wetland types. The y-axis lists AIC scores for each model centered on the mean AIC score of all the models. A lower centered AIC score for a model suggests better performance for that spatial scale and monthly lag combination. The background color shows the interpolated relative importance of a particular monthly lag averaged across all the models included in the plot. Red bands indicate monthly lags when hydrological conditions have the highest relative importance

Wetland explanatory variables were most strongly associated with *Cs. melanura* abundance at broad spatial scales ranging from 1000 m to 3000 m from mosquito trapping sites (Fig. 2, Additional file 2: Figure S1). However, emergent wetlands had an important relationship with vector abundance at 100 m, the second smallest spatial scale assessed (Additional file 2: Figure S1b). The relationship between human development and abundance was strongest within 200 m of trapping sites and progressively decreased in strength at broader spatial scales (Additional file 2: Figure S1f).

We found that changes in hydrological wetness during several time periods were important in explaining *Cs. melanura* abundance patterns. Hydrological wetness conditions 1 month before mosquito collection were significantly associated with *Cs. melanura* abundance (Fig. 2a). Hydrological wetness conditions 8 months before and during mosquito collection (0 month lag), also had strong relationships with abundance (Fig. 2b).

#### EEEV infection in *Cs. melanura*

Wetland explanatory variables also had spatial scale-dependent relationships with EEEV presence/absence in *Cs. melanura*. Deciduous forested wetland and emergent wetlands were most strongly associated with EEEV infection at broad scales (2000–5000 m) (Additional file 3: Figure S2a, b).

The timing of hydrological conditions was also important in explaining variation in EEEV infection. Hydrological wetness 10 months prior to sampling had the strongest association with EEEV infection (Fig. 2c), however an important relationship was also detected during the month of mosquito sampling (0 month lag) (Fig. 2c).

### Wetland effects on *Cs. melanura* abundance

The best performing GAM model explained 50.8% of the total deviance in monthly *Cs. melanura* abundance. Here we describe the modeled relationships between wetlands, hydrological wetness conditions, and the abundance of *Cs. melanura*. Full model results are presented in Table 2.

**Table 2:** Best-performing Generalized Additive Model (GAM) estimating *Cs*. *melanura* abundance. All explanatory variables included in the model are listed in the left-most column, including the most important spatial scale or temporal lag for that variable. The proportion of the deviance explained by each explanatory variable is listed in the right-most column. The proportion of the total deviance explained by the full model is listed in the left-most column header. For the sixth variable listed, there were two time periods when hydrological conditions were important. The variable for the second important time-period and the output values that change between models are listed in brackets

Response: Abundance (Deviance explained: 0.50)	*F*-score	*P*	Deviance explained
Emergent Wetland 100 m	103.8 [98.2]	< 0.001	0.01
Evergreen Forested Wetland 1000 m	170.5 [166.6]	< 0.001	0.07
Deciduous Forested Wetland 3000 m	408.8 [340.8]	< 0.001	0.13
Scrub/Shrub Wetland 3000 m	94.1	< 0.001	0.02
Stream Connectivity 2000 m	20.3 [20.5]	< 0.001	0.01
Forested Wetland 3000 m * PHDI (0 lag) OR [Forested Wetland 2000 m * PHDI (8 lag)]	10.1 [17.9]	< 0.001	0.01
Semi-permanent Wetland 3000 m * PHDI (1 lag)	72.9 [66.2]	< 0.001	0.02
Impervious Surfaces 200 m	213.7 [201.6]	< 0.001	0.11
Month	340.5 [309.75]	< 0.001	0.07
% Gravid Traps (linear)	-24.0 [22.7] (t)	< 0.001	0.04

#### Wetland vegetation

**Fig. 3:**
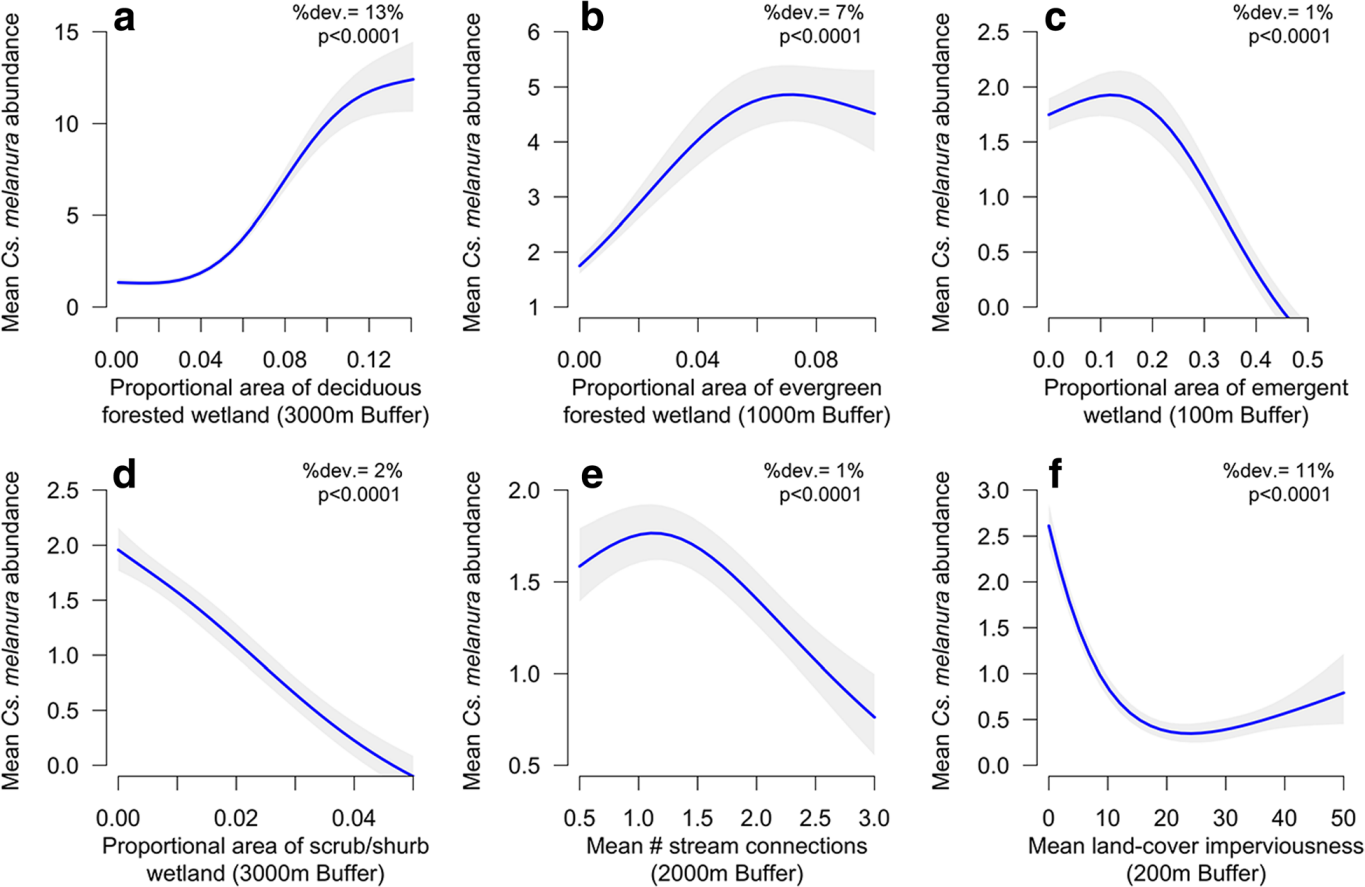
Generalized additive model (GAM) response curves depicting the relationship between mean *Cs. melanura* abundance per sampling night and 6 explanatory variables: (**a**) deciduous forested wetland; (**b**) evergreen forested wetland; (**c**) emergent wetland; (**d**) scrub/shrub wetland; (**e**) stream connectivity; and (**f**) impervious surfaces (Table 2). The percent of the total model deviance explained by each variable (%dev.) and associated *P*-values are listed in the upper right of each plot. Grey bands represent 95% confidence intervals (1.96*SE) on the estimated *Cs. melanura* abundance based on GAM predictions

Wetland relationships with *Cs. melanura* abundance varied depending on wetland vegetative characteristics. Wetlands dominated by deciduous forest were associated with greater abundances of *Cs. melanura* at nearby mosquito trapping sites (Fig. 3a). GAM model results indicate that trapping locations surrounded by a low proportional area of deciduous wetland (< 4% of area) captured an average of less than 3 *Cs. melanura* individuals per night, whereas sites with the highest proportional area of deciduous wetland (> 12% of area) captured 10 to 15 individuals (Fig. 3a). Evergreen forested wetlands were also associated with greater *Cs. melanura* abundance (Fig. 3b). However, the observed increase in abundance with increasing evergreen forested wetland area was less than for deciduous forested wetlands, with the expected abundance ranging from less than 2 *Cs. melanura* individuals per night at low levels of evergreen wetland (< 1% of area) to more than 4 individuals at the highest levels of evergreen wetland (> 8% of area) (Fig. 3b). Emergent- and shrub-dominated wetlands, which do not serve as larval habitat for *Cs. melanura,* had statistically significant negative relationships with abundance (Fig. 3c, d). High proportional areas of these wetland types resulted in approximately 2 fewer *Cs. melanura* captured per night than in locations with few of these wetlands (Fig. 3c, d).

#### Wetland connectivity and hydrology

**Fig. 4:**
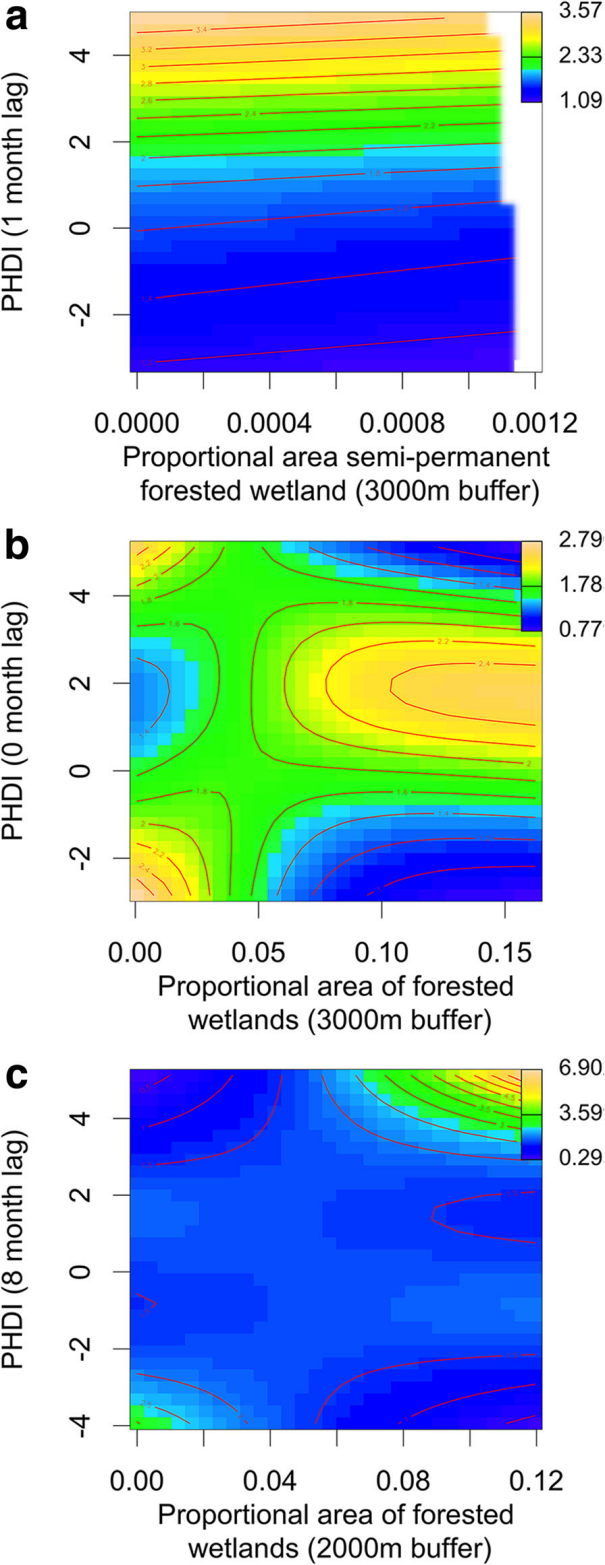
Contour plots showing the effects of interactions between the proportional area of forested semi-permanent wetland (**a**) or all types of forested wetlands (**b**, **c**), and hydrological conditions (PHDI) on *Cs. melanura* abundance. (**a**) and (**b**) show this relationship during the transmission season (0–1 month lag) and (**c**) depicts the previous fall/winter (8 month lag). Blue represents low vector abundance and yellow represents high abundance. The range of abundance values is listed in the upper right of each plot

Wetland connectivity and inundation classification/hydrological wetness were significantly associated with *Cs. melanura* abundance. A greater mean number of stream connections to forested wetlands was associated with a non-linear reduction in the observed abundance of *Cs. melanura* (Fig. 3e). No strong relationships were observed when forested wetlands were connected to fewer than 1.5 streams, but at greater levels of stream connectivity (~3 stream connections), vector abundance was lower (~1 individual per night) (Fig. 3e). Monthly average hydrological wetness also had significant relationships with *Cs. melanura* abundance (Fig. 4). Moderate to very wet hydrological conditions (PHDI > 1) 1 month before mosquito sampling were associated with greater vector abundances, regardless of semi-permanent forested wetland area (Fig. 4a). Dry (PHDI < −1) and extremely wet conditions (PHDI > 4) during the sampling month (0 month lag) in conjunction with a large quantity of forested wetlands were also associated with lower vector abundance, whereas moderately wet conditions (~1–3 PHDI) combined with forested wetlands were associated with higher abundance (Fig. 4b). Finally, extremely wet hydrological conditions (PHDI > 4) 8 months before mosquito sampling were associated with greater vector abundances, but only when all types of forested wetland were at their highest levels (> 10% of area) (Fig. 4c).

#### Wetland effects on EEEV infection in *Cs. melanura*

The best performing GAM model explained 49.3% of the total deviance in EEEV presence/absence. Here we describe wetland vegetation, size and inundation/hydrological wetness relationships with the log odds of EEEV presence in *Cs. melanura*. Detailed model results are available in Table 3.

**Table 3:** Best-performing Generalized Additive Model (GAM) estimating EEEV presence/absence in *Cs*. *melanura*. All explanatory variables included in the model are listed in the left-most column, including the most important spatial scale or temporal lag for that variable. The proportion of the deviance explained by each explanatory variable is listed in the right-most column. The proportion of the total deviance explained by the full model is listed in the left-most column header. For the sixth variable listed, there were two time periods when hydrological conditions were important. The variable for the second important time-period and the output values that change between models are listed in brackets

Response: EEEV Pres/Abs (Deviance explained: 0.49 [0.46])	*χ* ^2^	*P*	Deviance explained
Emergent Wetland 2000 m	8.2 [4.6]	0.01 [0.06]*	0.01
Evergreen Forested Wetland 1000 m	0 [1.4]	0.3 [0.1]	0
Deciduous Forested Wetland 5000 m	6.1 [15.6]	0.005 [< 0.001]*	0.03 [0.01]
Scrub/Shrub Wetland 5000 m	0 [0]	0.8 [1]	0
Average Forested Wetland Size 1500 m	2.1 [0.01]	0.08 [0.2]	0.01 [0.02]
Relative semi-permanent wetland 500 m* PHDI (10 lag) OR [Relative semi-permanent wetland 500 m* PHDI (0 lag)]	43.5 [24.7]	< 0.001*	0.06
Impervious Surfaces 200 m	3.7 [3.9]	0.1 [0.1]	0.01
Month	50.1 [51.6]	< 0.001*	0.17
Mean *Cs. melanura* abundance	70.9 [53.8]	< 0.001*	0.15
% Gravid Traps (linear)	-4.3 [−4.7] (z)	< 0.001*	0.05

*P < 0.05

#### Wetland vegetation

**Fig. 5:**
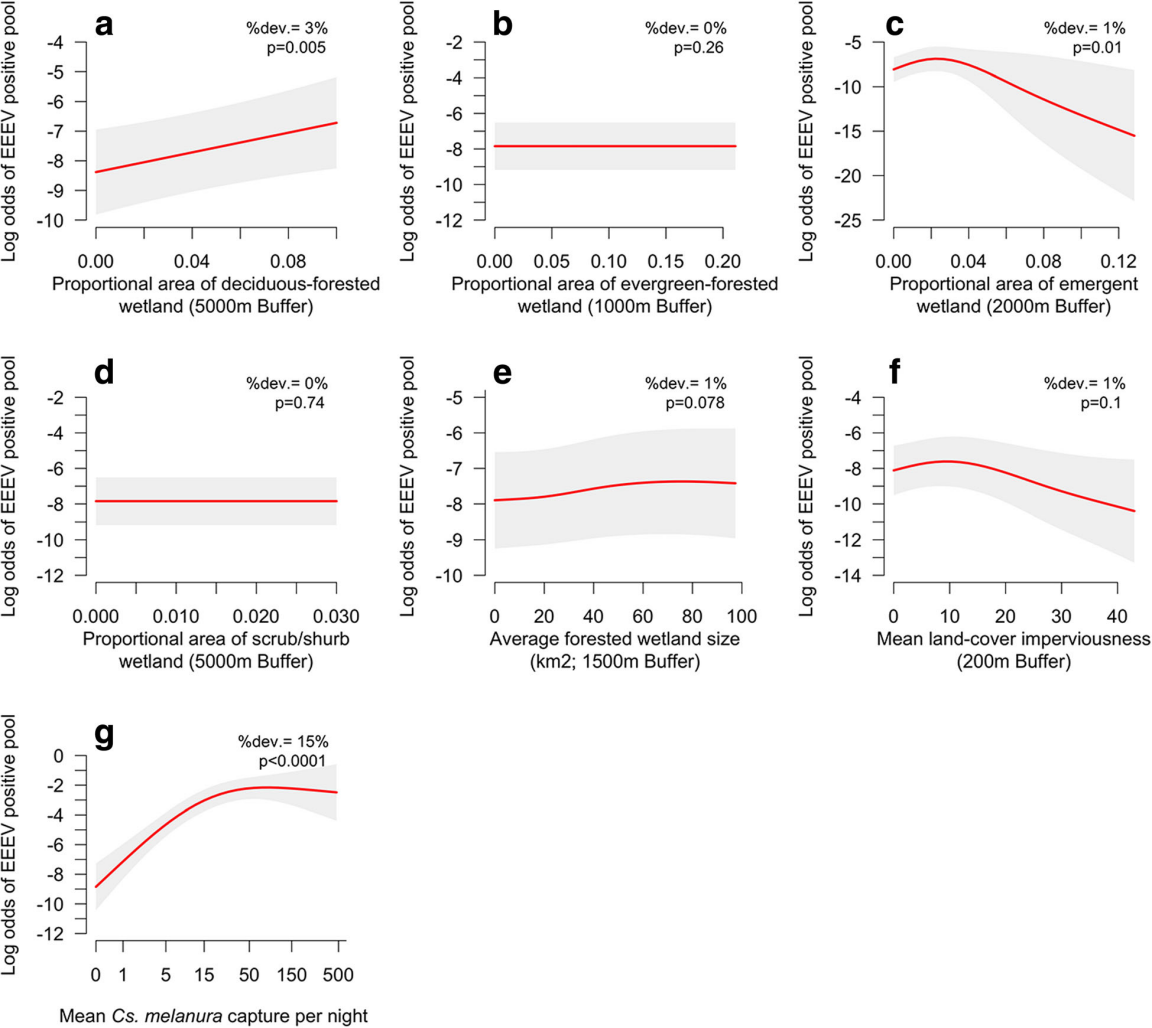
Generalized additive model (GAM) response curves depicting the relationship between the log odds of an EEEV positive *Cs. melanura* pool and 7 explanatory variables: (**a**) deciduous forested wetland; (**b**) evergreen forested wetland; (**c**) emergent wetland; (**d**) scrub/shrub wetland; (**e**) forested wetland size; (**f**) impervious surfaces; and (**g**) mean *Cs. melanura* abundance (Table 3). The percent of the total model deviance explained by each variable (%dev.) and associated *P*-values are listed in the upper right of each plot. Grey bands represent 95% confidence intervals (1.96*SE) on the estimated log odds of EEEV presence based on GAM predictions

Several wetland vegetative characteristics were associated with changes in the log odds of EEEV presence. However, the relationships between wetland vegetation and EEEV infection were weak relative to vegetation associations with *Cs. melanura* abundance. Wetlands dominated by deciduous forest had a nearly linear (*edf* = 0.83) positive association with the log odds of EEEV presence (Fig. 5a). Additionally, the proportional area of emergent wetlands had a weak but statistically significant negative association with the log odds of EEEV presence (Fig. 5c). Evergreen forested wetlands and scrub-shrub wetlands did not have statistically significant relationships with EEEV infection (Fig. 5b, d).

#### Wetland size and hydrology

**Fig. 6:**
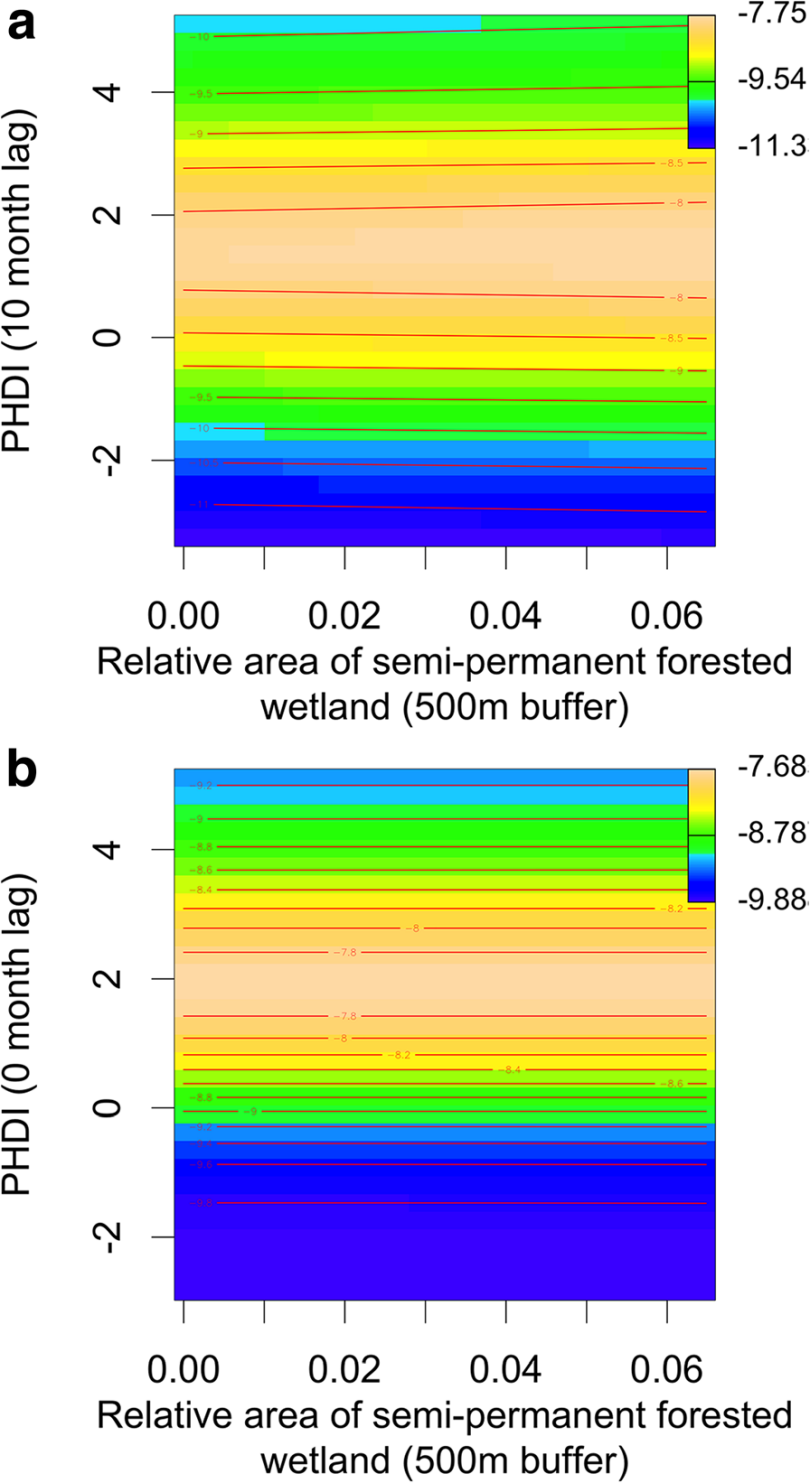
Contour plots showing the effects of interactions between the relative area of semi-permanent wetland and hydrological conditions (PHDI) on the log odds of an EEEV positive *Cs. melanura* pool. (**a**) shows this relationship during the previous fall/winter (10 month lag) and (**b**) depicts the relationship during the transmission season (0 month lag). Blue represents low log odds of EEEV presence, yellow represents high log odds of EEEV presence, and the value range is listed in the upper right of both plots

We found no statistically significant relationship between average forested wetland size and the log odds of EEEV presence (Fig. 5e). Moderately wet hydrological conditions (~1–3 PHDI) were linked with a higher log odds of EEEV presence during two separate time periods, 10 months before (Fig. 6a) and during mosquito sampling (0 month lag) (Fig. 6b). During these same time periods, dry hydrological wetness conditions (PHDI < -1) and very wet hydrological conditions (PHDI > 3) were associated with lower log odds of infection (Fig. 6a, b). These relationships were independent of the relative area of semi-permanent forested wetland (Fig. 6a, b).

## Discussion

Our aim was to identify broad-scale relationships between wetland characteristics and *Cs. melanura* abundance and infection with EEEV and the spatial scales and temporal periods over which these relationships are most important. Based on our spatial scale analysis, we conclude that the risks of EEEV transmission are spatially widespread, extending several kilometers from wetland habitats. Further, wetland vegetative characteristics had strong links with *Cs. melanura* abundance and weaker but significant associations with EEEV infection. Analyses of hydrologic and anthropogenic variables’ indicate that EEEV transmission may be severe in rural areas and when hydrological conditions are particularly wet during the summer transmission season and during the fall/winter before the transmission season. Overall, these results expand the local-scale understanding of the relationships between wetland characteristics and EEEV transmission to broad scales that more closely align with landscape-wide patterns in epizootic EEEV activity.

### Importance of spatial scales and temporal lags

We found that relationships between wetland characteristics and both vector abundance and EEEV infection varied depending on the spatial scale at which wetlands were measured. The strongest relationships for both response variables were generally detected when wetland characteristics were quantified at broad spatial scales with buffer radii ranging between 1000 m and 5000 m. Vector abundance may be most closely linked to broad-scale wetland characteristics because of the strong dispersal capabilities of *Cs. melanura*. Nulliparous female *Cs. melanura* readily disperse from larval habitats to upland areas [54] and have an estimated mean dispersal distance ranging from 4000 m to 9000 m [55]. In contrast, broad scale wetland characteristics may be important predictors of EEEV infection due to strong vector dispersal and the extensive movements of important blood hosts of *Cs. melanura* like the house sparrow and American robin [56] that frequently travel 1000–2000 m to nesting sites and foraging sites [57–60].

There were also several discrete time periods when hydrological conditions had important associations with both *Cs. melanura* abundance and EEEV infection. These time periods included the transmission season (0–1 months before the sampling month) and the fall/winter before the transmission season (8 or 10 months before sampling month). Hydrological conditions during these periods may influence both vector abundance and EEEV infection as a result of associated changes in the availability/suitability of *Cs. melanura* larval habitat, passerine habitat, and resulting contact between these taxa (see below for details) [21, 30–33].

### Wetland characteristics and *Cs. melanura* abundance

Our analysis revealed linkages between wetland vegetative and connectivity characteristics and *Cs. melanura* abundance. Of the wetland vegetative factors assessed, forested deciduous and forested evergreen wetlands were most strongly associated with *Cs. melanura* abundance and together explained 20% of the total deviance in *Cs. melanura* abundance. This result closely aligns with previous fine-scale research indicating that larval *Cs. melanura* proliferate in forested swamps [8, 15, 19]. Deciduous forested wetland explained a greater proportion of the model deviance and increases in the quantity of this wetland type were associated with more drastic increases in *Cs. melanura* abundance as compared to evergreen forested wetland. This result indicates that deciduous wetland may be a more important source of larval habitat in Connecticut.

Further, forested deciduous and evergreen wetlands exhibited a non-linear relationship with *Cs. melanura* abundance. The sigmoid shape of the deciduous wetland vs abundance response curve (Fig. 3a) indicates that there is minimum quantity of deciduous wetland before changes in abundance can be detected (~4% of land cover), and that there is a threshold after which additional increases in deciduous forested wetland results in little detectable change in abundance (~12% of land cover) at the 3000 m scale. The threshold relationship between evergreen wetlands and abundance (Fig. 3b) also indicates that there may be minimal additional positive effects on abundance once evergreen forested wetland covers more than ~7% of landscape at the 1000 m scale. The minimum threshold for deciduous wetland likely reflects issues with detectability of low *Cs. melanura* abundances at relatively broad spatial scales (3000 m). The maximum thresholds for both forested wetland types may indicate that female *Cs. melanura* disperse greater distances for host seeking when abundance is high, thus reducing local densities [6]. These broad scale thresholds are important because vector density may reflect epizootic EEEV risks [19].

Several other wetland factors were also associated with *Cs. melanura* abundance. The proportional area of emergent wetlands, scrub-shrub wetlands, and the number of stream connections in forested wetlands all had statistically significant negative relationships with abundance (Fig. 3c-e). Each of these factors may have a negative relationship with abundance because they can facilitate the dispersal of mosquito predators like larval amphibians, small fishes, and larval macroinvertebrate insects to nearby forested *Cs. melanura* habitats [20, 21]. However, these wetland characteristics only explained ~4% of the total model deviance, and there is limited field research substantiating the observed relationships with *Cs. melanura* abundance. Therefore, we cannot confirm that predator movement between aquatic habitats has an ecologically meaningful effect on *Cs. melanura* abundance. Further research is needed to examine the spatial and temporal distribution of *Cs. melanura* predators and how these patterns influence *Cs. melanura* habitat selection and abundance.

### Wetland characteristics and EEEV infection in *Cs. melanura*

Wetland characteristics were also associated with EEEV infection in *Cs. melanura*, although wetland variables altogether explained only 11% of the total model deviance. Deciduous wetland had a linear positive relationship with EEEV infection in *Cs. melanura* (Fig. 5a). This indicates that forested wetlands may be focal points for transmission because they attract high densities of birds, including a variety of susceptible passerines, to *Cs. melanura* larval habitat containing abundant sources of food, water, and nesting habitat [10]. Evergreen, emergent vegetation, scrub-shrub vegetation, and the size of forested wetlands had either non-significant or very weak relationships with EEEV infection in *Cs. melanura* (Fig. 5b-e, Table 3).

Although direct links between wetlands and EEEV infection appear to be weak, indirect links may be substantial. We found that *Cs. melanura* abundance had a strong positive non-linear relationship with EEEV infection and was one of the most important explanatory variables (Fig. 5g). As detailed in the previous section, wetland vegetation strongly influenced vector abundance. Therefore, there may be important indirect effects of wetland vegetation on EEEV infection due to changes in *Cs. melanura* abundance. The threshold detected (Fig. 5g) suggests that positive effects of *Cs. melanura* abundance on EEEV infection may taper off when mean capture reaches around 15 individuals per night and that abundances of *Cs. melanura* above this level may not promote more intense viral amplification. However, we cannot rule out that associations between *Cs. melanura* abundance and EEEV infection are related to increases in the likelihood of detecting EEEV in mosquito pools rather than real changes in EEEV infection rates. Nevertheless, previous studies have concluded that *Cs. melanura* abundance is in fact a key facet in determining the risk of epizootic EEEV activity [19, 61]. It would be valuable for future studies to evaluate the effects wetlands on EEEV infection rates in *Cs. melanura* rather than EEEV presence/absence, in order to account for the effect of abundance on the likelihood of detecting EEEV. Future studies would also benefit from better estimates of the distribution and abundance of key EEEV avian hosts, in order to identify the habitats where contact between vectors and hosts is most likely to occur.

### Broader hydrological and anthropogenic context of EEEV transmission

Our models examined temporal changes in hydrological wetness conditions (measured via PHDI), how this factor interacts with the wetland inundation classification, and the resulting changes in *Cs. melanura* abundance and infection with EEEV. Findings indicate that when inundation levels are high in forested wetlands during the transmission season, there is likely a greater quantity of available larval habitat and vector abundance increases. Summer inundation of forested wetlands has been previously linked to increases in *Cs. melanura* abundance [6]. Our results also suggest that fall/winter precipitation improves *Cs. melanura* overwintering survival in subterranean crypts where above and below-ground water levels are influenced by water table depth [2, 11]. In contrast, we found that hydrological conditions were not important when forested wetland was rare (Fig. 4b, c), suggesting that groundwater conditions have less influence on the availability of *Cs. melanura* habitat in upland locations or in wetlands with other vegetative characteristics.

Relationships between hydrological wetness and EEEV infection were independent of the relative area of semi-permanent forested wetland (Fig. 6a, b). Consequently, we conclude that the contraction of wetland area during drought does not result in increased contact between vectors and hosts as observed for West Nile virus and St. Louis encephalitis virus in Florida [30–33]. Instead, we hypothesize that wet conditions during the fall/winter and during the transmission season promote the proliferation of available food, nesting resources, or an expansion of home ranges for susceptible-bird species and may increase brood size or offspring survival, leading to an influx of immunologically naïve fledglings that facilitate viral amplification [62, 63]. Moderately wet fall/winter hydrological conditions may also be important for EEEV infection because they may improve the survival of altricial birds, including passerines [64, 65]. However, it appears that extremely wet conditions are associated with lower EEEV infection, which could be related to documented declines in passerine recruitment and parental survival when conditions are very wet [63, 66, 67]. Future studies should examine how wet conditions influence tradeoffs between the positive effects of more food resources on avian recruitment and the negative influence of rainfall on parental nest visitation.

The importance of the effects of wetland characteristics on *Cs. melanura* abundance is shaped by the level of local-scale human development (200 m from sampling location). We identified sharp decreases in *Cs. melanura* abundance as local urbanization increased from nearly no human development (0% impervious surfaces) to 20% coverage with impervious surfaces (Fig. 3f) - a level of development associated with a mix of lawns, aesthetic vegetation, and large-lot residential housing [68]. When the percentage of impervious surface coverage was greater than 20%, further urbanization had minimal effects on *Cs. melanura* abundance (Fig. 3f). These relationships were independent of nearby wetland characteristics. This indicates that even when there are forested wetlands in anthropogenic settings, including locations with dense residential housing, fewer *Cs. melanura* will be present than in comparable rural settings. Anthropogenic development did not have a significant relationship with EEEV infection. Therefore, susceptible avian hosts likely do not show strong preferences for rural or developed habitats, but risks of epizootic EEEV transmission may still be highest in rural locations near wetlands where *Cs. melanura* is most abundant.

## Conclusions

Overall, wetland characteristics, particularly wetland vegetation, were important in determining spatial patterns in *Cs. melanura* abundance and, to a lesser extent, *Cs. melanura* EEEV infection. Additionally, our identification of the key spatial scales and temporal lags influencing *Cs. melanura* abundance and EEEV infection highlights the importance and utility of explicit evaluations of scale and timing for understanding mosquito-borne disease transmission. Future studies should also quantify wetland characteristics at broad scales and at multiple time periods to more precisely identify the location and timing of EEEV outbreaks within the northeastern US.

## Additional files

Additional file 1: Method of spatial scale and temporal lag selection. (DOCX 123 kb)

Additional file 2: Figure S1. Relative importance of spatial scales from 50 m to 5000 m for **a** mean number of stream connections to forested wetlands, **b** proportional area of emergent wetland, **c** proportional area of deciduous forested wetland, **d** proportional area of evergreen forested wetland, **e** proportional area of scrub/shrub wetland and **f** mean impervious surface coverage. Each point represents a different model explaining *Cs. melanura* abundance. The y-axis lists AIC scores for each model centered on the mean AIC score of all the models. A lower centered AIC score for a model suggests better performance for that spatial scale. The background color shows the interpolated relative importance of a particular spatial scale averaged across all the models included in the plot. Red bands indicate spatial scales where the explanatory variable has the highest relative importance. (DOCX 2196 kb)

Additional file 3: Figure S2. Relative importance of spatial scales from 50 m to 5000 m for **a** proportional area of deciduous forested wetland, and **b** proportional area of emergent wetland (only statistically significant explanatory variables included). Each point represents a different model explaining the presence/absence of EEEV infection in *Cs. melanura*. The y-axis lists AIC scores for each model centered on the mean AIC score of all the models. A lower centered AIC score for a model suggests better performance for that spatial scale. The background color shows the interpolated relative importance of a particular spatial scale averaged across all the models included in the plot. Red bands indicate spatial scales where the explanatory variable has the highest relative importance. (DOCX 709 kb)

## Abbreviations

EEEV: Eastern equine encephalitis virus
GAMs: Generalized Additive Models
NHD: National Hydrography Dataset
NLCD: National Land Cover Database
NWI: National Wetlands Inventory
PHDI: Palmer Hydrological Drought Index
SLEV: St. Louis encephalitis virus
WNV: West Nile virus
