# Supplementary material for: Wetland characteristics linked to broad-scale patterns in Culiseta melanura abundance and eastern equine encephalitis virus infection
Source: Parasit Vectors. 2017 Oct 18;10:501. doi: 10.1186/s13071-017-2482-0 (PMC5648514; doi:10.1186/s13071-017-2482-0)
Supplement: Supplementary file 1 — Method of spatial scale and temporal lag selection. (DOCX 123 kb) [file 13071_2017_2482_MOESM1_ESM.docx]

**Additional File 1: Method of Spatial Scale and Temporal Lag Selection**

Adapting a previously developed method [1], we first used Generalized Additive Models (GAMs) to individually regress each covariate with each response at every spatial scale or temporal lag. For example, when a spatial variable (e.g. deciduous forested wetland) was measured at 11 buffer sizes, there were 11 regression models for that variable for each response variable. In some cases, we could not include the smallest spatial scales for a wetland covariate in the GAM models because too few of the sampling sites had that type of wetland in their vicinity for there to be enough non-zero data to run the GAM model. Of all the univariate models generated, we selected those containing scales likely to have the best fit in the context of the full model. We accomplish this by selecting models with an AIC score that is 1.) lower than a model that includes only the non-spatial/non-temporal covariates, 2.) lower than the AIC of models with the next higher scale and next lower scale, and 3.) at least 2 AIC units lower than the model with the highest (worst) AIC score. To identify the best scales/lags in interactions between hydrological wetness conditions and wetland area, a similar approach was employed. The AIC of models with the interactive term had to be 1.) lower than a model that includes only non-spatial/non-temporal covariates, 2.) at least 2 units lower than maximum AIC score of all other models containing either the scale or lag included in the interaction (i.e. lower than all other interactions with the same spatial scale, and also lower than all other interactions with the same monthly lag), and 3.) lower than interaction terms containing the next higher and next lower scale/lag for both variables in the interaction. For instance, to include an interaction between a spatial and temporal covariate, the AIC had to be lower than other interaction terms with the same spatial scale but with temporal lags 1 month earlier and 1 month later and the AIC score also had to be lower than interaction terms with the same temporal lag, but with the next smaller and next larger spatial scale. The goal of this step was to remove irrelevant spatial scales and temporal lags from further consideration [1].

We then ran GAMs on all combinations of the remaining covariate scales in a single model, testing only the scales and lags selected above to determine which combinations provided the best fit in the context of a full model. We identified particularly important scales/lags among all the tested models by calculating relative variable importance. This technique uses Akaike weights to rank the importance of a variable relative to other variables [2]. We adapted this metric to summarize the relative importance of lags and scales by 1.) calculating Akaike weights for all models with the same response variable, 2.) summing the weights across all of these models with the same scale or lag, and 3.) calculating the relative importance by dividing the summed weights for the scale/lag with the summed weights of all the models. We then linearly interpolated these importance scores across a continuous range of potential scales/lags using the *stats* package in R 3.2.3 to approximate importance values at the scales/lags intervening the discrete scales/lags that were evaluated. Through this process, we identified the most important scales/lags for each explanatory variable.

1. Bradter U, Kunin WE, Altringham JD, Thom TJ, Benton TG. Identifying appropriate spatial scales of predictors in species distribution models with the random forest algorithm. Methods in Ecology and Evolution. 2013;4(2):167-74.

2. Burnham KP, Anderson DR. Model selection and multimodel inference: A practical information-theoretic approach, 2nd ed: Springer Science & Business Media; 2003.
