# Supplementary material for: Wetland characteristics linked to broad-scale patterns in Culiseta melanura abundance and eastern equine encephalitis virus infection
Source: Parasit Vectors. 2017 Oct 18;10:501. doi: 10.1186/s13071-017-2482-0 (PMC5648514; doi:10.1186/s13071-017-2482-0)
Supplement: Supplementary file 2 — Relative importance of spatial scales from 50 m to 5000 m for a mean number of stream connections to forested wetlands, b proportional area of emergent wetland, c proportional area of deciduous forested wetland, d proportional area of evergreen forested wetland, e proportional area of scrub/shrub wetland and f mean impervious surface coverage. Each point represents a different model explaining Cs. melanura abundance. The y-axis lists AIC scores for each model centered on the mean AIC score of all the models. A lower centered AIC score for a model suggests better performance for that spatial scale. The background color shows the interpolated relative importance of a particular spatial scale averaged across all the models included in the plot. Red bands indicate spatial scales where the explanatory variable has the highest relative importance. (DOCX 2196 kb) [file 13071_2017_2482_MOESM2_ESM.docx]

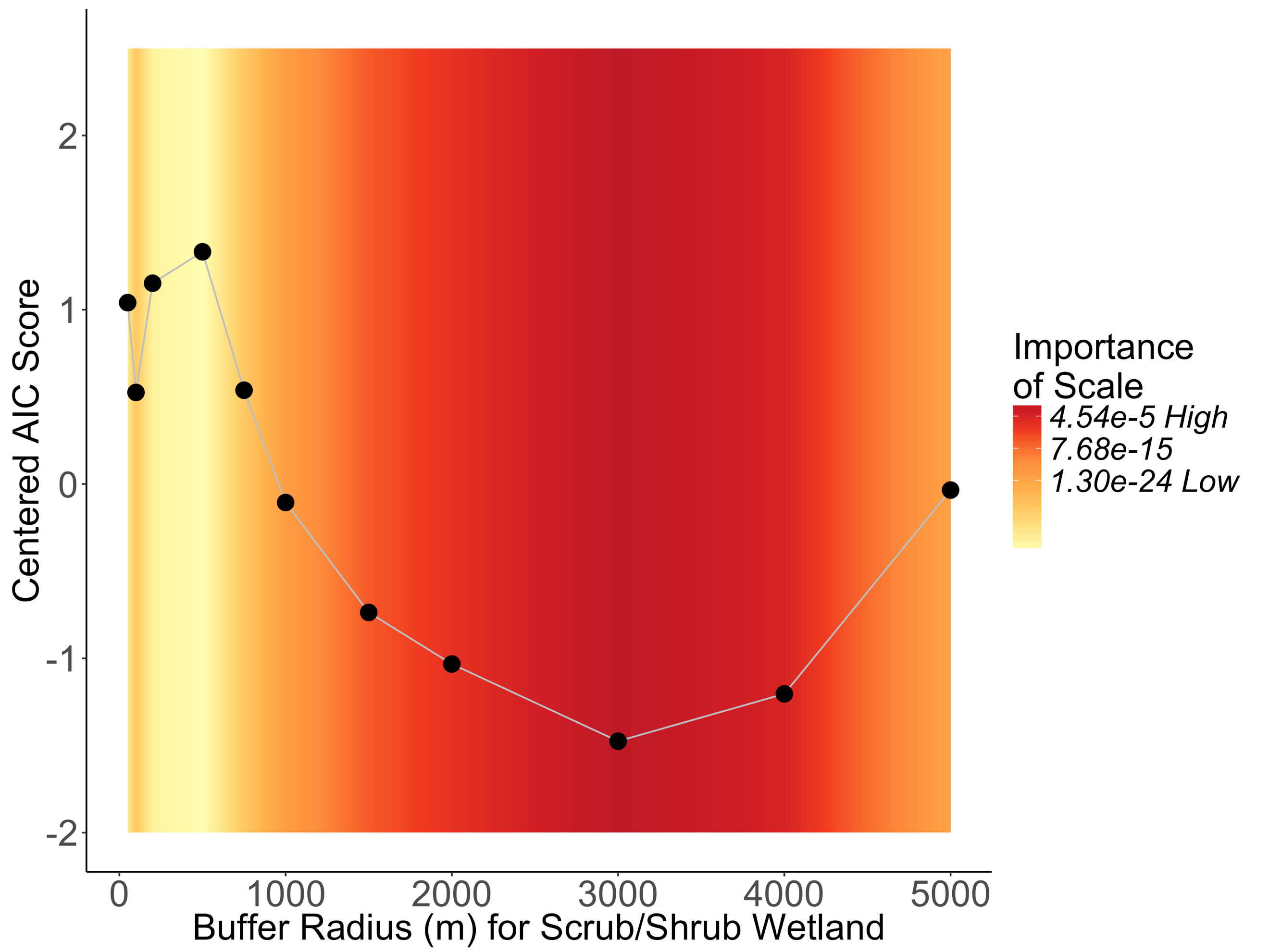

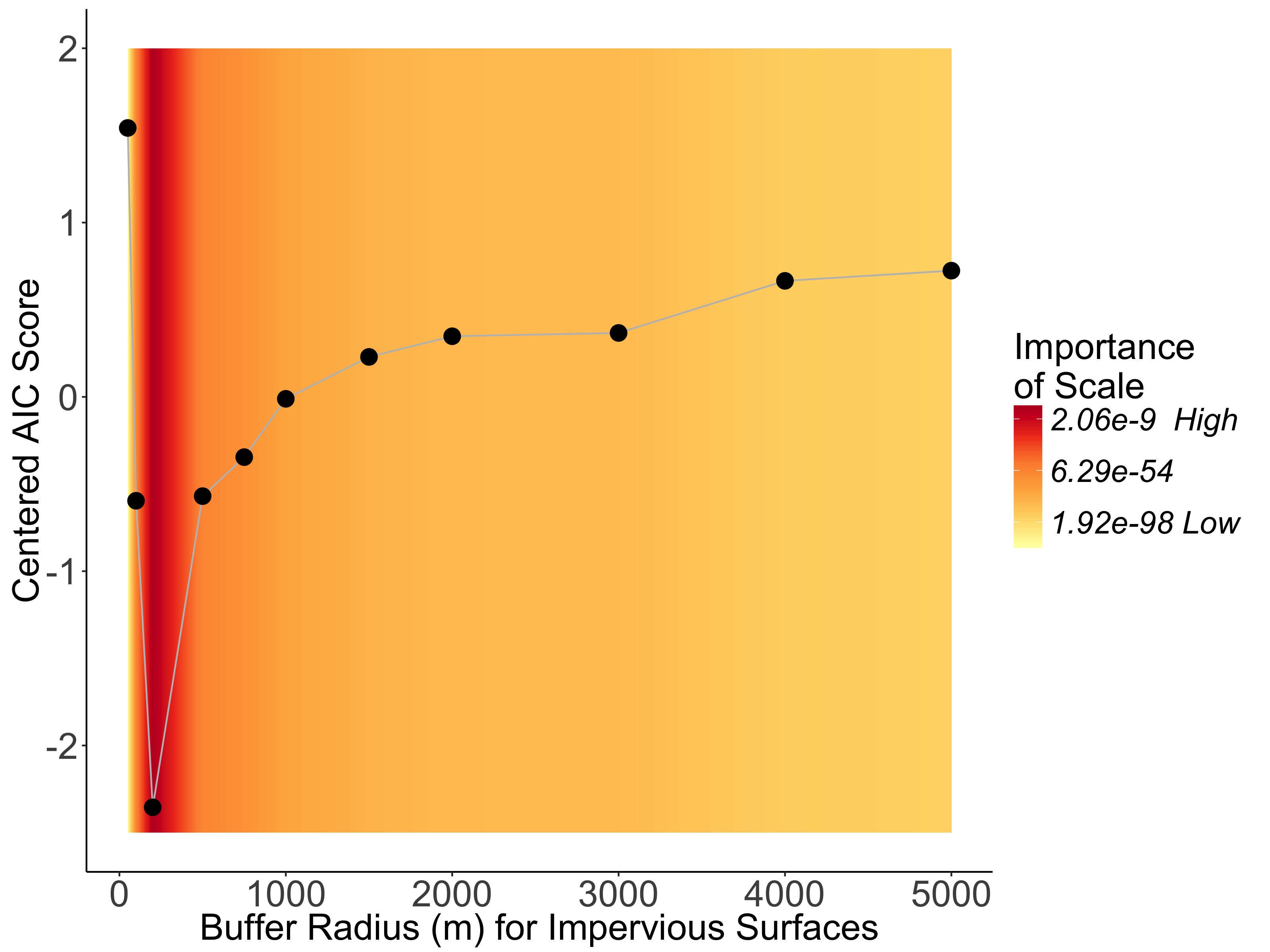

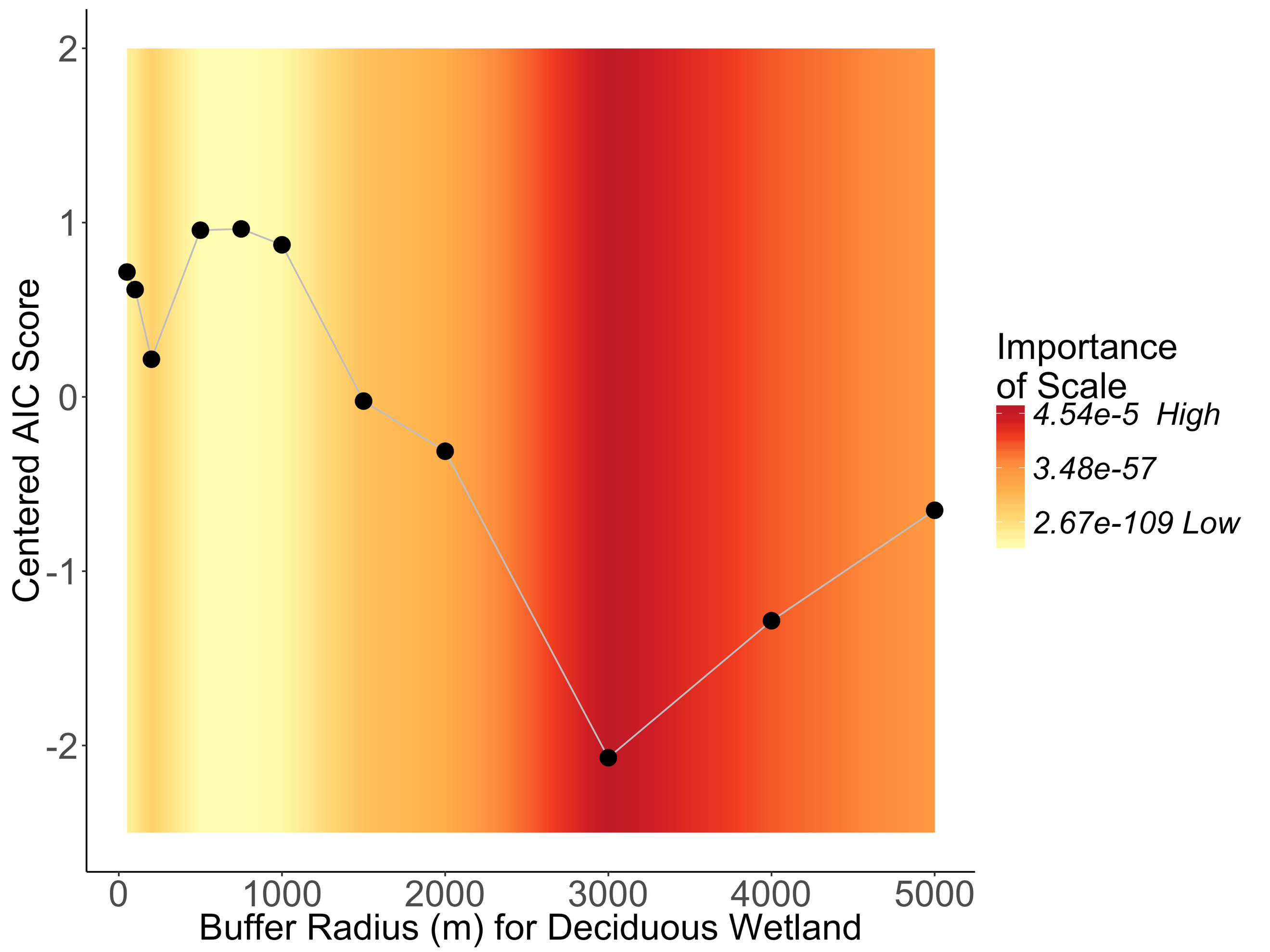

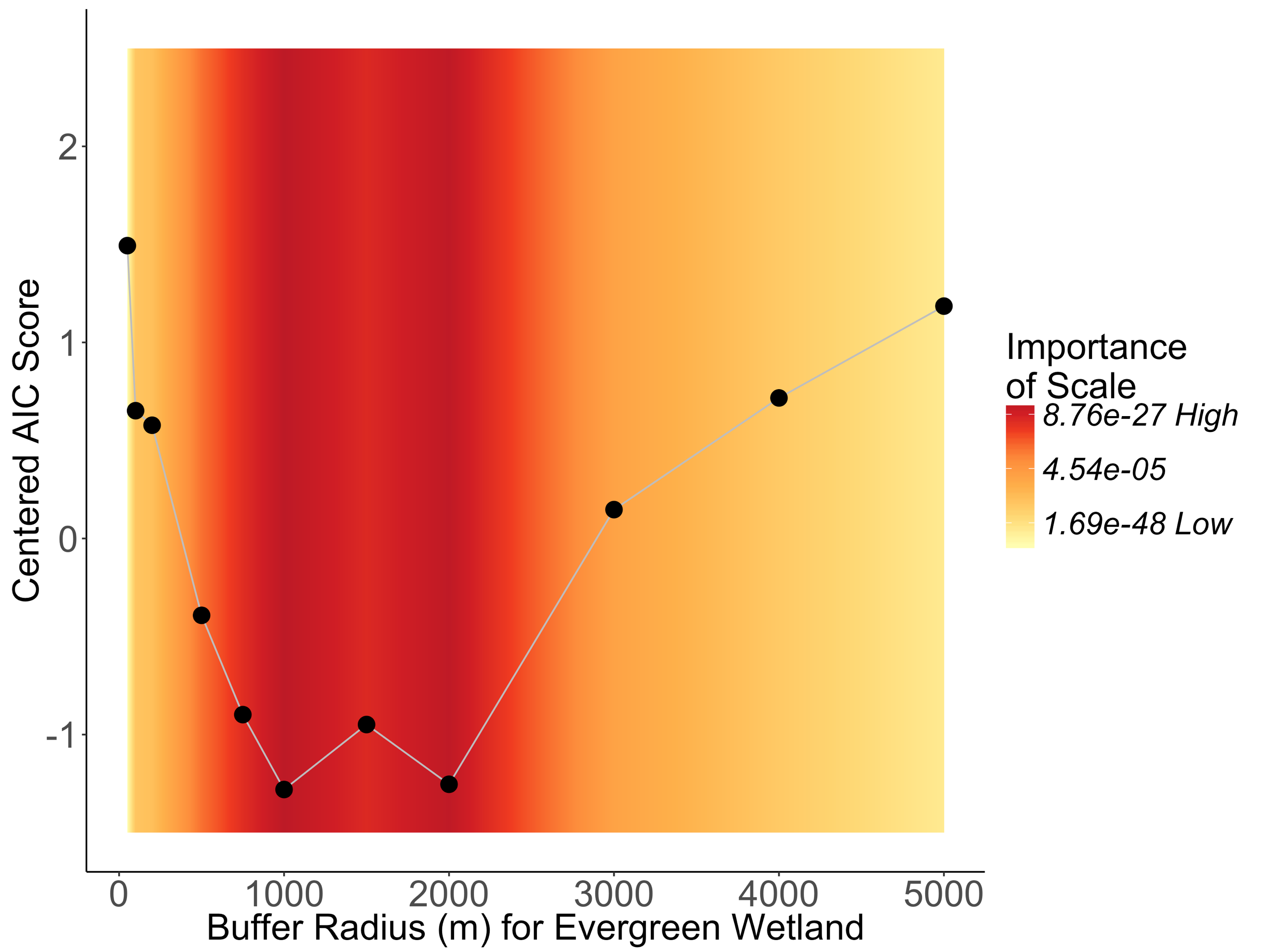

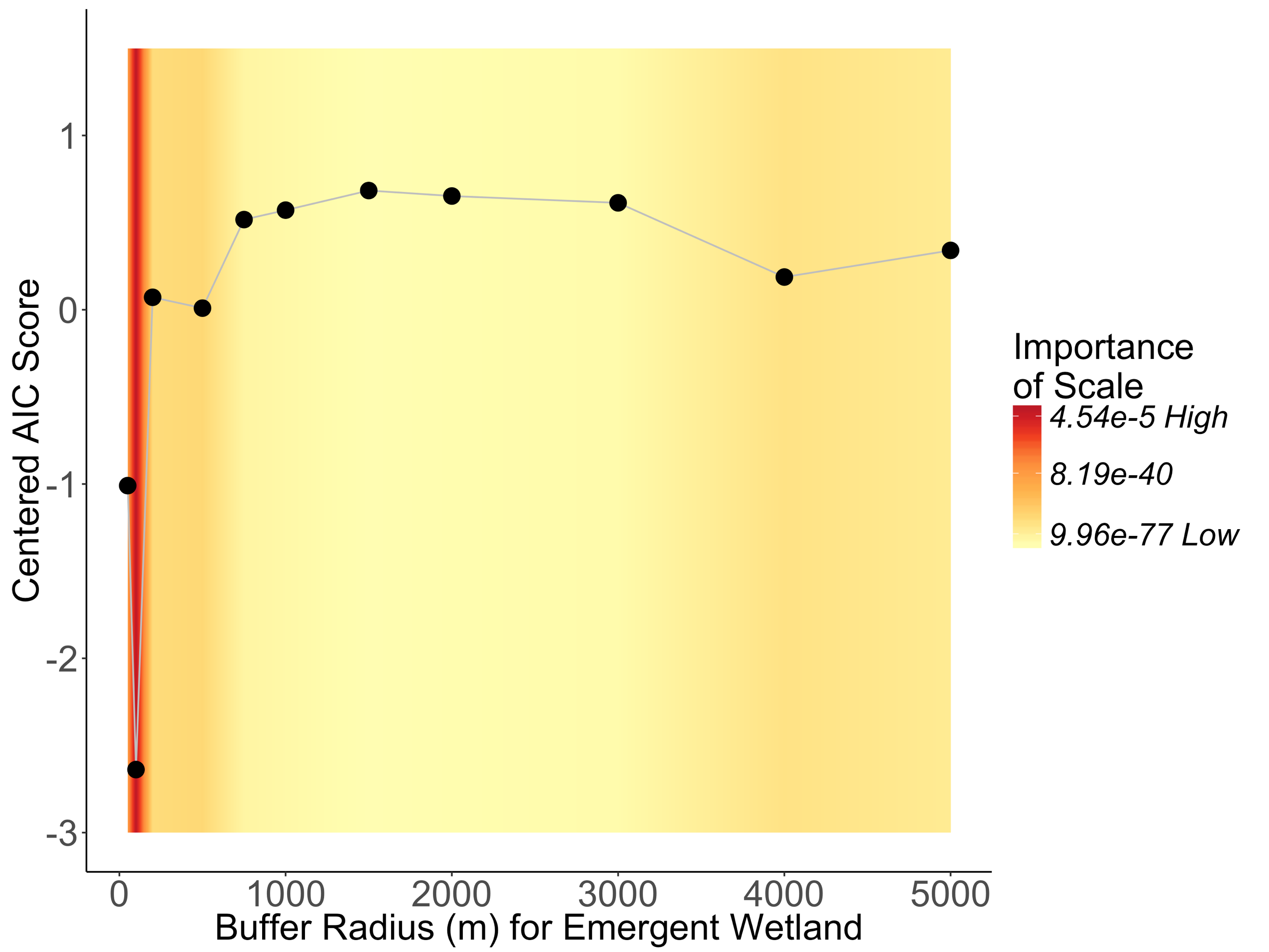

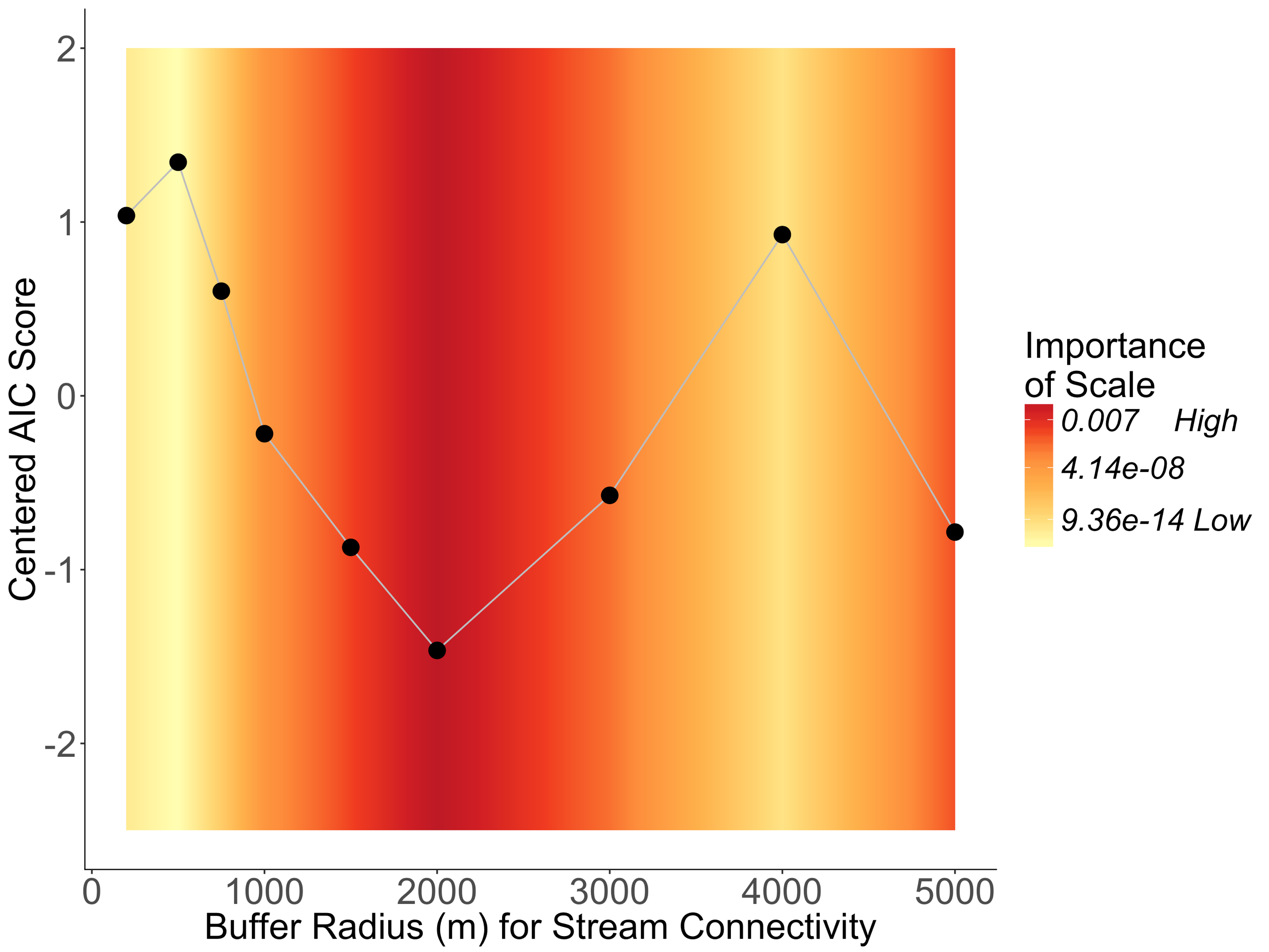


**Fig. S1:** Relative importance of spatial scales from 50 m to 5000 m for **a** mean number of stream connections to forested wetlands, **b** proportional area of emergent wetland, **c** proportional area of deciduous forested wetland, **d** proportional area of evergreen forested wetland, **e** proportional area of scrub/shrub wetland and **f** mean impervious surface coverage. Each point represents a different model explaining *Cs. melanura* abundance. The y-axis lists AIC scores for each model centered on the mean AIC score of all the models. A lower centered AIC score for a model suggests better performance for that spatial scale. The background color shows the interpolated relative importance of a particular spatial scale averaged across all the models included in the plot. Red bands indicate spatial scales where the explanatory variable has the highest relative importance

f

e

d

b

a

c
