# Supplementary material for: Wetland characteristics linked to broad-scale patterns in Culiseta melanura abundance and eastern equine encephalitis virus infection
Source: Parasit Vectors. 2017 Oct 18;10:501. doi: 10.1186/s13071-017-2482-0 (PMC5648514; doi:10.1186/s13071-017-2482-0)
Supplement: Supplementary file 3 — Relative importance of spatial scales from 50 m to 5000 m for a proportional area of deciduous forested wetland, and b proportional area of emergent wetland (only statistically significant explanatory variables included). Each point represents a different model explaining the presence/absence of EEEV infection in Cs. melanura. The y-axis lists AIC scores for each model centered on the mean AIC score of all the models. A lower centered AIC score for a model suggests better performance for that spatial scale. The background color shows the interpolated relative importance of a particular spatial scale averaged across all the models included in the plot. Red bands indicate spatial scales where the explanatory variable has the highest relative importance. (DOCX 709 kb) [file 13071_2017_2482_MOESM3_ESM.docx]

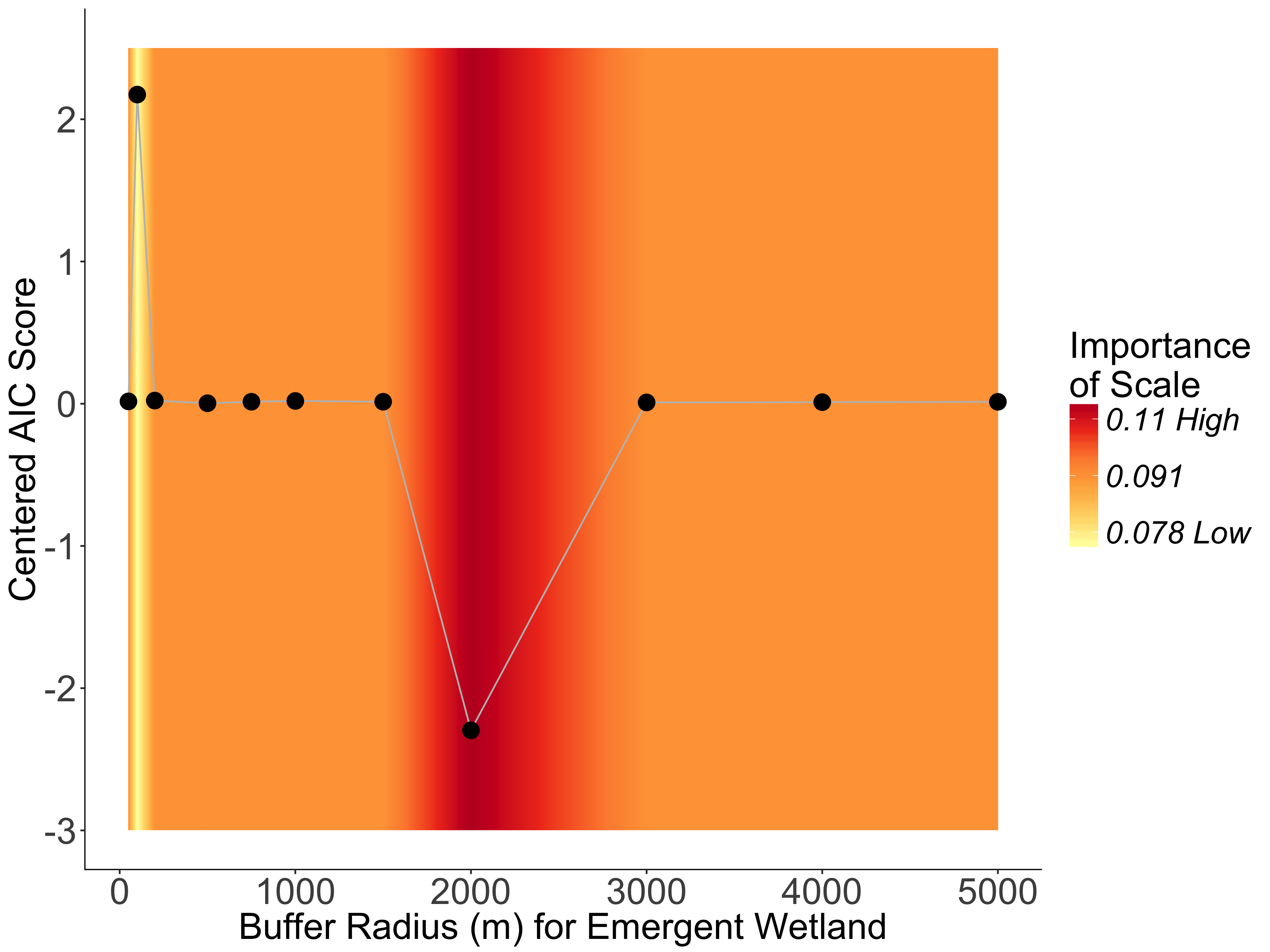

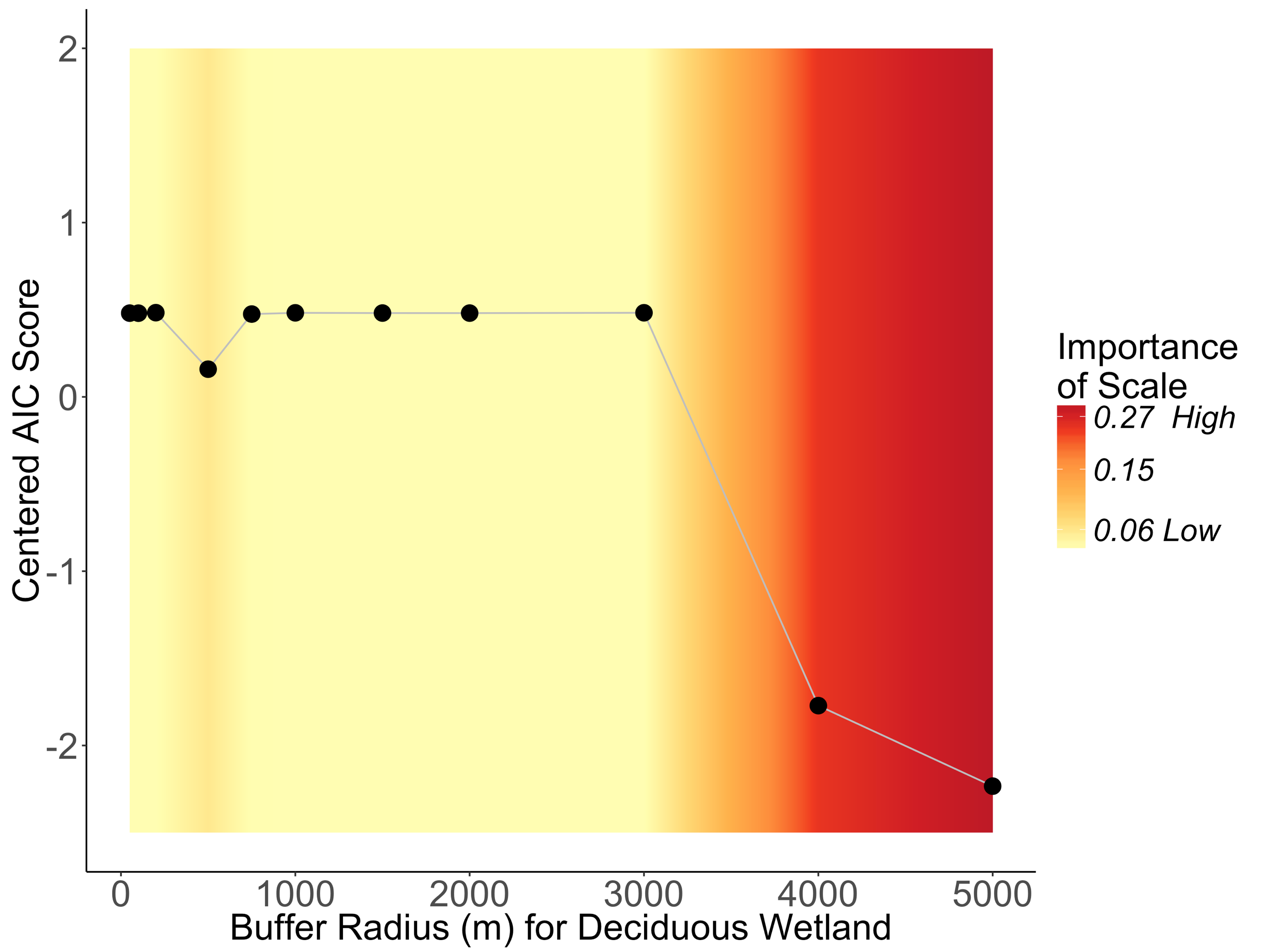


**Fig. S2:** Relative importance of spatial scales from 50 m to 5000 m for **a** proportional area of deciduous forested wetland, and **b** proportional area of emergent wetland (only statistically significant explanatory variables included). Each point represents a different model explaining the presence/absence of EEEV infection in *Cs. melanura*. The y-axis lists AIC scores for each model centered on the mean AIC score of all the models. A lower centered AIC score for a model suggests better performance for that spatial scale. The background color shows the interpolated relative importance of a particular spatial scale averaged across all the models included in the plot. Red bands indicate spatial scales where the explanatory variable has the highest relative importance

a

b
